# A systematic review for the evidence of recommendations and guidelines in hybrid nuclear cardiovascular imaging

**DOI:** 10.1007/s00259-024-06597-x

**Published:** 2024-01-15

**Authors:** Florent L. Besson, Giorgio Treglia, Jan Bucerius, Constantinos Anagnostopoulos, Ronny R. Buechel, Marc R. Dweck, Paula A. Erba, Oliver Gaemperli, Alessia Gimelli, Olivier Gheysens, Andor W. J. M. Glaudemans, Gilbert Habib, Fabian Hyafil, Mark Lubberink, Christopher Rischpler, Antti Saraste, Riemer H. J. A. Slart

**Affiliations:** 1 Department of Nuclear Medicine-Molecular Imaging, DMU SMART IMAGING, Hôpitaux Universitaires Paris-Saclay, AP-HP, CHU Bicêtre, Le Kremlin Bicetre, France; 2 School of Medicine, Université Paris-Saclay, Le Kremlin-Bicetre, France; 3 Commissariat À L’énergie Atomique Et Aux Énergies Alternatives (CEA), Centre National de La Recherche Scientifique (CNRS), Inserm, BioMaps, Université Paris-Saclay, Le Kremlin-Bicetre, France; 4 Division of Nuclear Medicine, Imaging Institute of Southern Switzerland, Ente Ospedaliero Cantonale, 6501 Bellinzona, Switzerland; 5 Faculty of Biomedical Sciences, Università della Svizzera Italiana, 6900 Lugano, Switzerland; 6 Department of Nuclear Medicine, Georg-August University Göttingen, Universitätsmedizin Göttingen, Gottingen, Germany; 7 BRFAA - Biomedical Research Foundation Academy of Athens, Athens, Greece; 8 Department of Nuclear Medicine, Cardiac Imaging, University Hospital Zurich, Zurich, Switzerland; 9 British Heart Foundation Centre for Cardiovascular Science, Edinburgh Heart Centre, University of Edinburgh, Chancellors Building, Little France Crescent, Edinburgh, UK; 10 Department of Medicine and Surgery, University of Milan Bicocca, and Nuclear Medicine Unit ASST Ospedale Papa Giovanni XXIII, Bergamo, Italy; 11 HeartClinic, Hirslanden Hospital Zurich, Zurich, Switzerland; 12 Fondazione Toscana/CNR Gabriele Monasterio, Pisa, Italy; 13 Department of Nuclear Medicine, Cliniques Universitaires Saint-Luc, Institut Roi Albert II, Université Catholique de Louvain, 1200 Brussels, Belgium; 14 Department of Nuclear Medicine and Molecular Imaging, Medical Imaging Center, University Medical Center Groningen, University of Groningen, Groningen, the Netherlands; 15 Department of Cardiology, APHM, La Timone Hospital, Marseille, France; 16 Department of Nuclear Medicine, DMU IMAGINA, Georges-Pompidou European Hospital, Assistance Publique - Hôpitaux de Paris, F75015 Paris, France; 17 Medical Imaging Centre, Uppsala University Hospital, Uppsala, Sweden; 18 Department of Nuclear Medicine, University Hospital Stuttgart, Stuttgart, Germany; 19 Heart Center, Turku University Hospital and University of Turku, Turku, Finland; 20 Department of Biomedical Photonic Imaging, Faculty of Science and Technology, University of Twente, Enschede, the Netherlands

**Keywords:** Positron emission tomography, Hybrid imaging, Cardiovascular guidelines, Recommendations, Evidence-based practice

## Abstract

**Objectives:**

This study aimed to evaluate the level of evidence of expert recommendations and guidelines for clinical indications and procedurals in hybrid nuclear cardiovascular imaging.

**Methods:**

From inception to August 2023, a PubMed literature analysis of the latest version of guidelines for clinical hybrid cardiovascular imaging techniques including SPECT(/CT), PET(/CT), and PET(/MRI) was performed in two categories: (1) for clinical indications for all-in primary diagnosis; subgroup in prognosis and therapy evaluation; and for (2) imaging procedurals. We surveyed to what degree these followed a standard methodology to collect the data and provide levels of evidence, and for which topic systematic review evidence was executed.

**Results:**

A total of 76 guidelines, published between 2013 and 2023, were included. The evidence of guidelines was based on systematic reviews in 7.9% of cases, non-systematic reviews in 47.4% of cases, a mix of systematic and non-systematic reviews in 19.7%, and 25% of guidelines did not report any evidence. Search strategy was reported in 36.8% of cases. Strengths of recommendation were clearly reported in 25% of guidelines. The notion of external review was explicitly reported in 23.7% of cases. Finally, the support of a methodologist was reported in 11.8% of the included guidelines.

**Conclusion:**

The use of evidence procedures for developing for evidence-based cardiovascular hybrid imaging recommendations and guidelines is currently suboptimal, highlighting the need for more standardized methodological procedures.

## Introduction

The number of guidelines released for positron emission tomography (PET) and PET/computed tomography (CT) has significantly increased in the past decade, and this trend is expected to continue growing with the increasing clinical applications of novel tracers and the adoption of new imaging modalities. In addition to the guidelines developed by highly respected professional organizations on major topics, numerous guidelines have been formulated by national and regional organizations or even expert panels to address specific questions relevant to local practice. While this diversity of guidelines aims to cater to the needs of different populations, it also raises concerns about the reliability of recommendations.

Guidelines from various specialties have consistently exhibited low methodological quality and inconsistent recommendations [1, 2], with some even failing to meet basic methodological standards [3]. Surprisingly, no methodological studies have been conducted for guidelines pertaining to PET, PET/CT, or PET/magnetic resonance imaging (MRI). The collection and utilization of evidence are fundamental processes that significantly impact the quality of guideline development and, consequently, the formulation of essential recommendations [4]. Among all forms of evidence, systematic reviews are considered the gold standard for guideline development worldwide [5]. This emphasis on developing recommendations based on systematic review evidence is also underscored in the definition of guidelines by the Institute of Medicine (IOM), which defines clinical practice guidelines as statements that include recommendations intended to optimize patient care, informed by a systematic review of evidence and an assessment of the benefits and harms of alternative care options [6]. Nevertheless, the extent to which systematic review evidence is used in developing recommendations for nuclear imaging guidelines remains unknown, or may not be properly executed.

The purpose of our study was to comprehensively review all clinical and technical practice guidelines, recommendations, and expert opinions related to hybrid nuclear cardiovascular imaging and available on PubMed. Our aim was to evaluate the extent to which these adhere to standardized methodologies for evidence collection and utilization. We also sought to identify areas within recommendation topics where systematic review evidence may be inadequately referenced.

## Methods

This was a cross-sectional systematic survey of published literature that did not involve human subjects, and hence was exempt from institutional review board approval.

### Selection of guidelines

We included international clinical practice guidelines published in peer-reviewed journals indexed in PubMed for the indications or procedures of SPECT(/CT), PET(/CT), or PET(/MRI) imaging applied to the following fields of cardiovascular imaging: heart failure, coronary artery disease, extra-cardiac atherosclerosis, infection (endocarditis, cardiovascular implantable electronic devices) and inflammation (large vessel vasculitis), amyloidosis, sarcoidosis, cardiotoxicity, radiation dose and safety, and artificial intelligence. For all topics, only guidelines published in English language were retained. Were considered as “guidelines”: (i) the publications which were self-identified as “guideline” and were developed or endorsed by official international consortiums or (ii) recommendations, high level position, and expert papers by experts in the fields; (iii) a single document may exist of more than one cardiovascular topic and counted additionally.

### Search of guidelines

For any of the fields of cardiovascular imaging, we searched PubMed from inception to August 2023 to identify guidelines pertaining to cardiovascular hybrid SPECT(/CT), PET(/CT), or PET(/MRI) for the diagnosis, treatment monitoring, or procedurals using the combination of the following terms: “((PET*) OR (positron) OR (SPECT*) OR (hybrid)) AND ((guideline*) OR (recommendation*) OR (position) OR (expert) OR (consensus)) AND ((*imaging procedure*) AND ((*disease*) AND (*diagnosis*/therapy evaluation OR monitoring OR treatment/prognosis OR event* OR survival OR follow up))).” In all cases, two predefined investigators dedicated to a field of interest and blinded to each other screened the documents retrieved by this search. Final results were centralized and checked by two investigators (R. H. J. A. Slart and F. L. Besson). Any disagreements were resolved by consensus with the investigators dedicated to the field of interest.

### Two categories of guidelines

All included guidelines were classified into two categories as follows: (1) guidelines for clinical purpose (i.e., diagnosis and/or therapy evaluation) and (2) guidelines for procedures, if providing step-by-step instructions for cardiovascular hybrid imaging.

### Survey of the collection and use of evidence

**Fig. 1:**
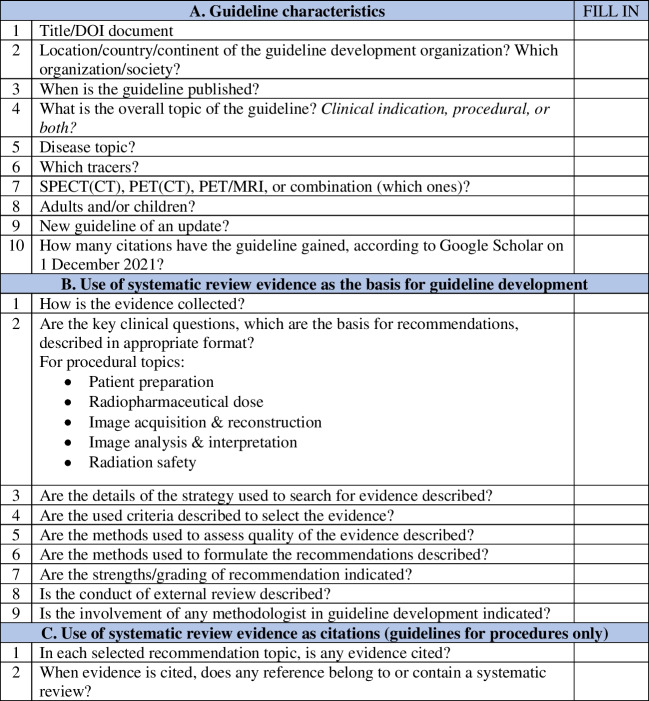
Questionnaire for the collection and use of evidence for guidelines

To survey the use of scientific evidence in the guidelines, we developed a questionnaire (Fig. 1) adapted from the article of Li et al. [7]. For each field of interest, the two pre-defined investigators surveyed the guidelines and cross-validated their findings. Disagreements were resolved by consensus. In the case of procedural guidelines, the following recommendation topics were searched and had to be fulfilled: patient preparation, radiopharmaceutical dosage, acquisition and reconstruction of images, analysis and interpretation of images, and radiation safety.

### Analysis

We conducted a qualitative analysis of the data providing absolute frequencies and proportions in % or median (IQR) when appropriate. Data analysis and visualization were conducted using Microsoft Excel (version 16.77.1).

## Results

**Fig. 2:**
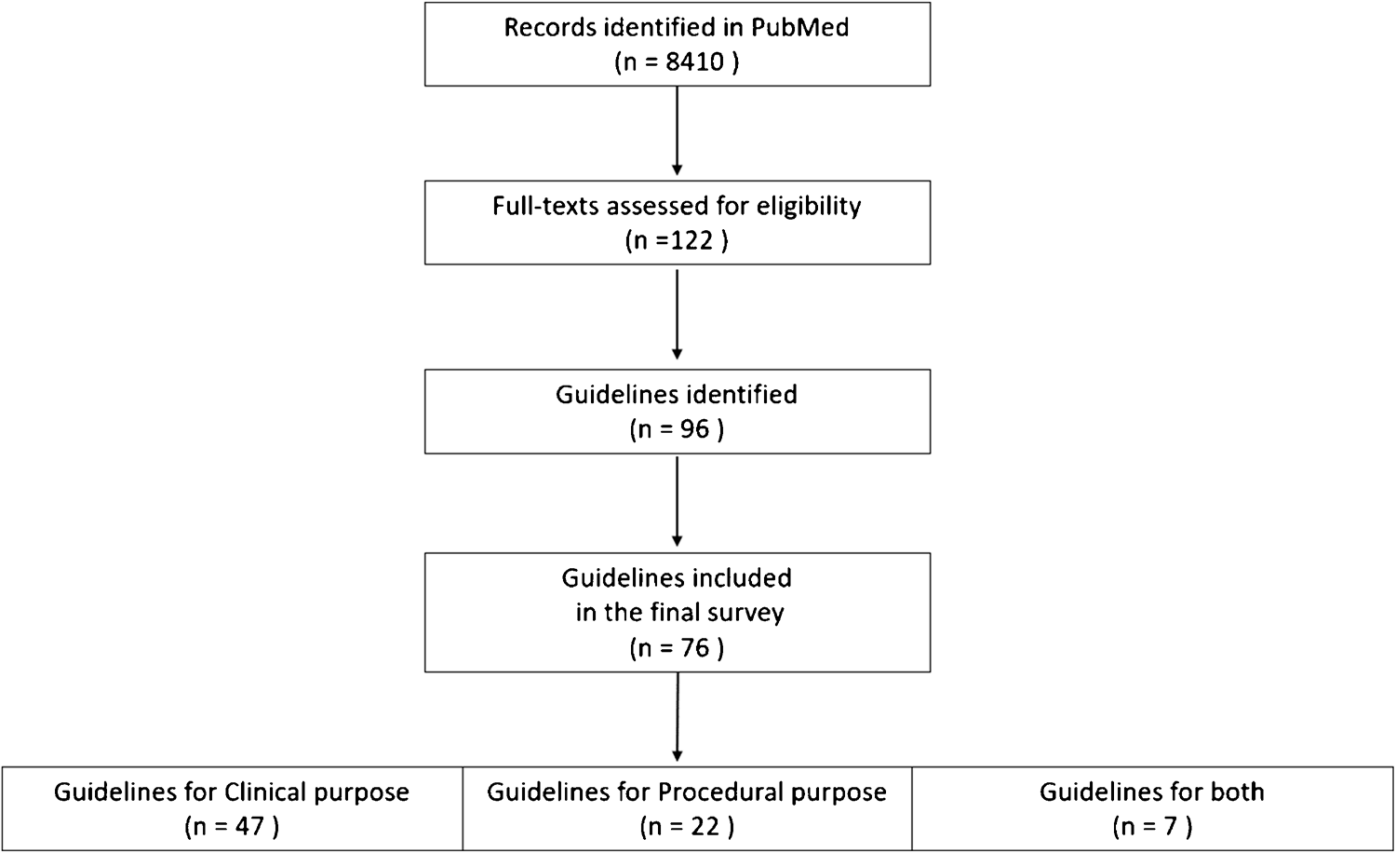
Flow diagram for literature screening

The overall flow diagram for literature screening is provided in Fig. 2. The overall search identified 76 records as guidelines for cardiovascular hybrid imaging.

### Characteristics of the included guidelines

**Fig. 3:**
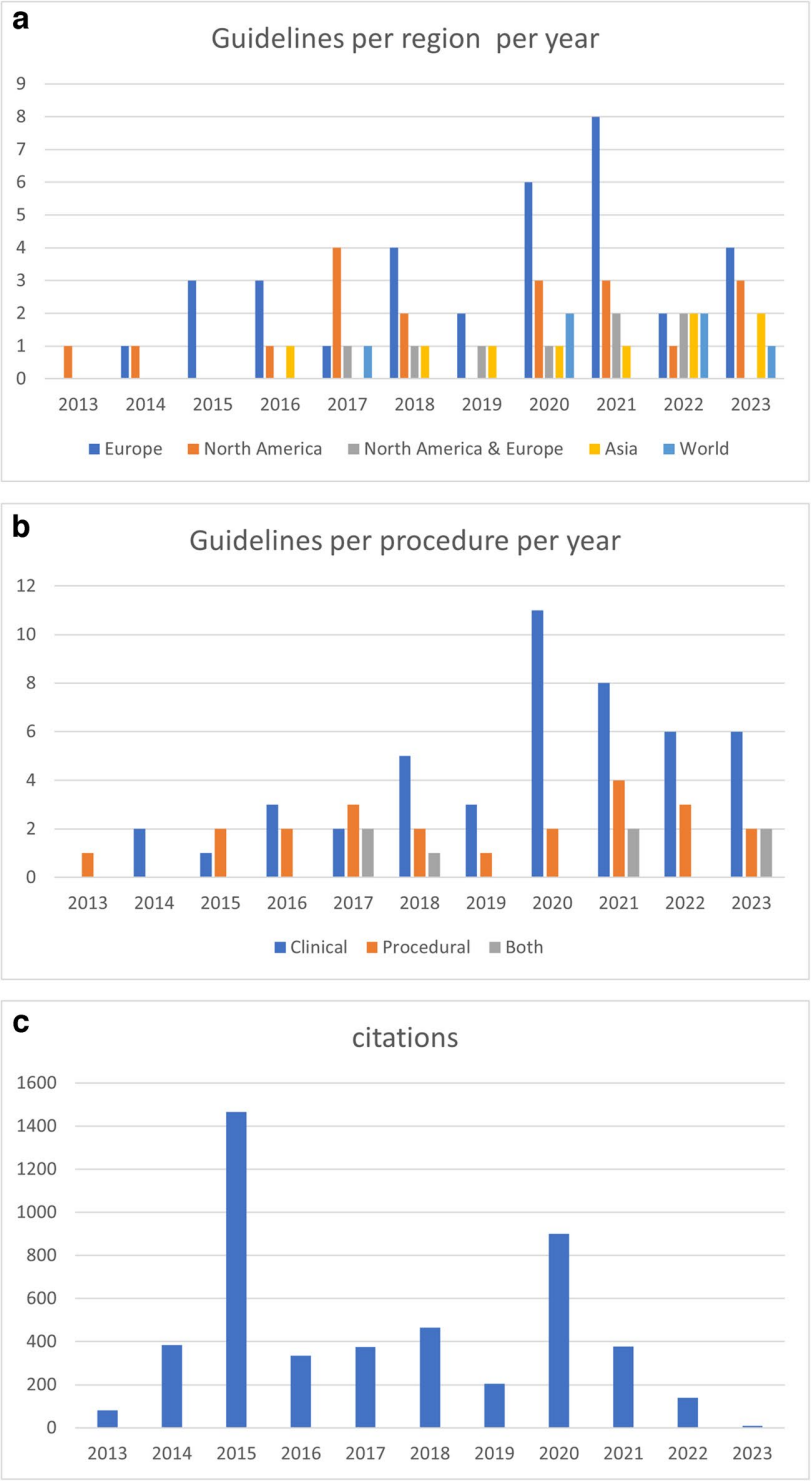
General characteristics

The main characteristics of the guidelines are provided in the Table 1 and Fig. 3. The included guidelines were published between 2013 and 2023, with a rate per year ranging from 1 (2013) [8] to 13 (2021) [9–21]. A total of 47 (62%) guidelines concerned clinical purposes [9, 11, 13–16, 20–60], whereas 22 (29%) guidelines concerned procedures [8, 10, 12, 17, 18, 61–75] and seven (9%) concerned both [10, 19, 76–80]. Three out of these papers existed of two different guidelines and counted double [10, 18, 61].

**Table 1:** Characteristics of the practice guidelines (*n* = 76)

Characteristics	*N*	Proportion
Regions		
• Europe • North America • Europe and North America • Asia • World consortiums	34 19 8 9 6	44.7% 25% 10.5% 11.8% 8%
Years of publication		
• 2013–2015 • 2016–2018 • 2019–2021 • 2022–2023	6 20 31 19	8% 26.3% 40.7% 25%
Contents		
• Clinical purpose • Procedural purpose • Both	47 22 7	61.8% 29% 9.2%
Field		
• Amyloidosis • CAD • Sarcoidosis • HF • IE/CIED • LVV • Atherosclerosis • Radiation dose and safety • Non obstructive CAD • Reporting • Cardiotoxicity • Takotsubo • LV dysfunction • Cardiomyopathy • Artificial intelligence	18 13 11 7 7 5 3 2 2 2 2 1 1 1 1	23.7% 17.1% 14.5% 9.2% 9.2% 6.6% 3,9% 2.6% 2.6% 2.6% 2.6% 1.3% 1.3% 1.3% 1.3%
Modality		
• SPECT(/CT) • PET/CT-MRI • General (both)	17 31 28	22.4% 40.8% 36.8%
Population		
• Adult • Children • Both • Unspecified	57 1 9 9	75% 1% 12% 12%
Citations		
• < 50 • 50–150 • > 150	56 14 6	73.7% 18.4% 7.9%

A total of 56 guidelines were identified as “original” [9–11, 13, 15–19, 21–30, 32, 34–39, 41–48, 50–53, 55, 56, 59–64, 67–70, 73, 75–77, 79, 80], 14 as “updates” [8, 14, 20, 31, 33, 40, 49, 57, 58, 65, 66, 71, 74, 78], and the remaining four where summaries [18, 54, 72] or reprint [12]. We included 28 guidelines for PET(/CT-MRI) [10, 14, 15, 18, 22, 23, 25, 26, 30–32, 34, 41, 47–49, 51, 52, 55, 57, 61–63, 66, 67, 69, 71, 78], 28 guidelines for both PET(/CT) and SPECT(/CT) [8, 11–13, 19, 20, 24, 27–29, 33, 35, 37, 39, 43, 45, 50, 53, 54, 56, 58–60, 64, 73, 75–77], and 17 guidelines for SPECT(/CT) [9, 16, 17, 21, 36, 38, 40, 42, 44, 46, 65, 68, 70, 72, 74, 79, 80]. Amyloidosis was the most represented condition (*n* = 18) [9, 11–13, 16–18, 21, 36, 38, 40, 42, 44, 46, 68, 72, 79, 80], followed by CAD (*n* = 13) [8, 20, 25, 34, 48, 53–55, 60, 61, 70, 74, 78], sarcoidosis (*n* = 11) [14, 22, 26, 30, 41, 57, 58, 62, 66, 71, 75], heart failure (*n* = 7) [27, 28, 35, 37, 45, 50, 56], CIED (*n* = 7) [10, 18, 33, 39, 49, 63, 64], LVV (*n* = 5) [10, 15, 31, 32, 69], extra-cardiac atherosclerosis (*n* = 3) [47, 52, 61], radiation dose and safety (*n* = 2) [76, 77], non-obstructive CAD (*n* = 2) [23, 43], cardiotoxicity (*n* = 2) [51, 59], reporting [67, 73], AI (*n* = 1) [19], gated blood pool assessment for LV dysfunction (*n* = 1) [65], cardiomyopathy (*n* = 1) [24], and Takotsubo (*n* = 1) [29]. The radiotracers explicitly concerned were mostly ^99m^Tc-based for SPECT (*n* = 29 guidelines) [8, 9, 11–14, 16, 17, 21, 24, 35, 36, 38, 40, 42, 44–46, 50, 58, 59, 65, 68, 70, 72–74, 79, 80] and ^18^F-FDG for PET (*n* = 37) [10, 14, 15, 18, 22, 24, 26–35, 39, 41, 45, 47–52, 57–59, 61–64, 66, 69, 71, 73, 75]. Also mentioned were ^123^I-mIBG for SPECT (*n* = 8) [27–29, 42, 45, 50, 59, 73] and ^82^Rb for PET (*n* = 8) [8, 14, 28, 35, 45, 59, 73, 78]. Other radiotracers including radiolabeled white blood cell, ^201^Thallium, ^11^C-based, ^13^N, ^18^F-Na, ^68^ Ga-somatostatin analogues, and ^67^ Ga were less frequently mentioned. The majority of the overall guidelines were developed by organizations located in western geographical areas (*n* = 64), of whom 31 from Europe [9–11, 16, 18, 19, 21, 23, 24, 27, 28, 31–34, 37, 39, 41, 43, 45, 47, 50, 51, 53, 54, 56, 59, 61, 64, 73, 74], 19 from North America [8, 14, 15, 20, 22, 25, 26, 30, 35, 36, 40, 49, 62, 67, 68, 71, 77, 78, 80], and 14 from mixed collaborations [12, 13, 29, 38, 46, 48, 52, 55, 63, 65, 69, 72, 75, 76]. The remaining of the guidelines were produced by Asian expert societies (*n* = 9) [17, 42, 44, 57, 58, 60, 66, 79], and six guidelines concerned broad international consortiums [38, 48, 52, 72, 76]. Near 75% of the guidelines concerned adult population (*n* = 57), one guideline dealing on radiation dose and safety focused exclusively on a pediatric population [76], and nine guidelines did not mention any targeted population [10, 27, 28, 37, 45, 50, 56, 63]. Only one paper assigned an appropriateness score for using nuclear imaging in the therapy management of the disease [13]; 12 other publications just mentioned nuclear imaging as an possible option [11, 24, 33, 41, 42, 57, 62, 65, 66, 68, 69, 75]. To note, the number of citations per guideline was highly heterogeneous, ranging from 0 ([14, 35, 44, 48, 60, 79] to 1337 of a previous clinical guideline [39], with a median number of citations per guideline of 18.5 (IQR = 4–59). Over the past 10 years (2013–2023), the cumulative citation rate per year ranged from 9 (2023) to 1465 (2015), with a median number of cumulative citations per year of 375 (IQR = 171.5–425).

### Quality assessment of the guidelines: evidence, search strategy, strength of recommendation, external review, and methodology

**Fig. 4:**
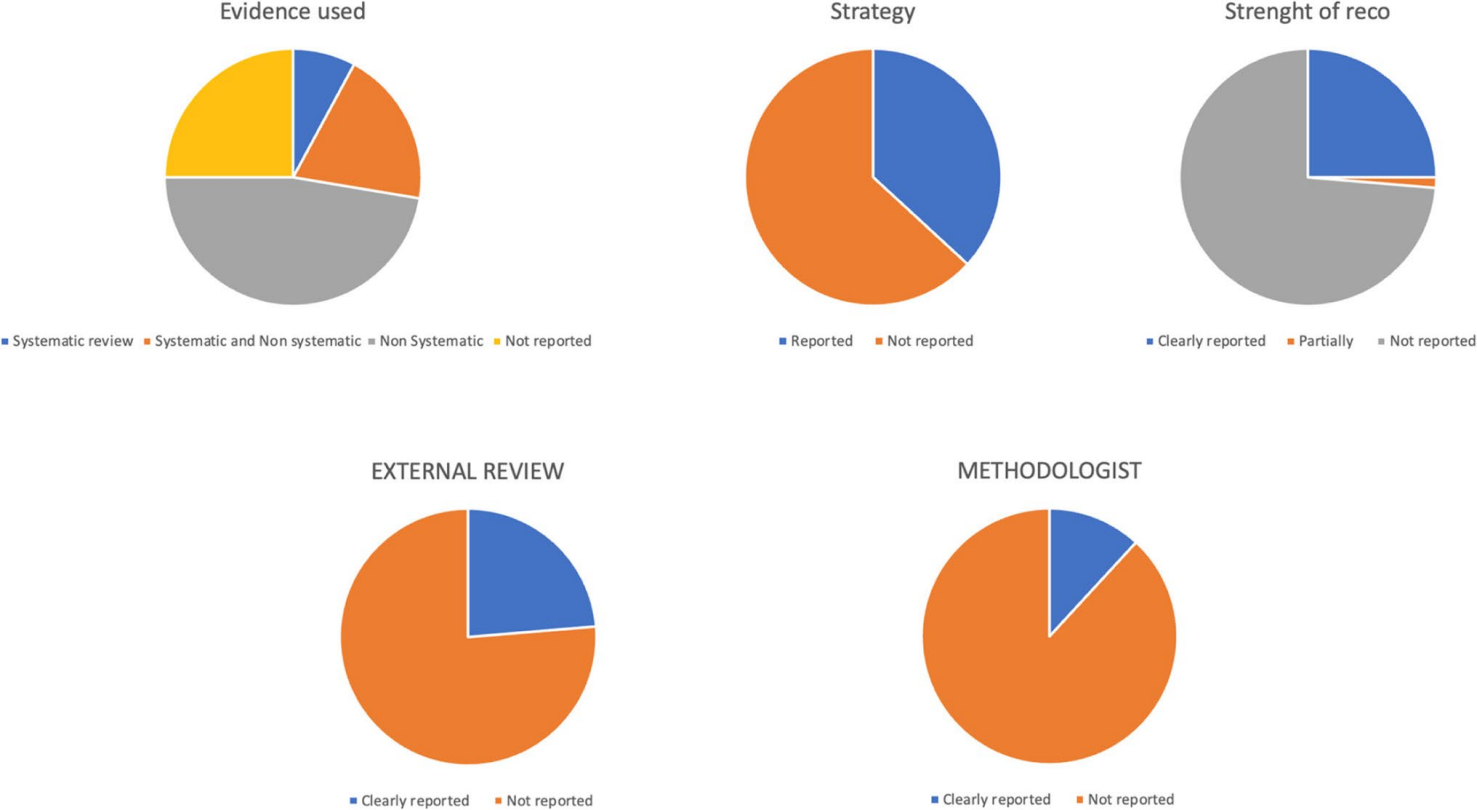
Quality assessment of the guidelines

The quality assessment of the included guidelines is provided in Table 2 and Fig. 4. The evidence was based on non-systematic reviews mainly (47.4%), followed by mix-based systematic and non-systematic reviews (19.7%) and systematic reviews (7.9%). In 25% of the cases, no evidence was mentioned. A search strategy was clearly reported in 28 guidelines (36.8%) [8, 12–15, 20, 22, 24, 26, 30–35, 37, 39, 40, 49, 58, 60–62, 66, 69, 71, 74, 75]. The strengths of recommendations were clearly reported in 19 guidelines (25%) [11, 14, 15, 20, 22, 26, 31, 32, 35–37, 40, 42, 49, 58, 60], partially reported in one guideline [69], and not reported in the remaining 56 guidelines (73.7%) [9, 10, 12, 16–19, 21, 27, 28, 30, 38, 44–48, 52, 56, 57, 63, 66, 72, 79, 80]. An external review was reported in 18 guidelines (23.7%) [8, 14, 15, 20, 22, 26, 30–32, 35, 37, 40, 42, 56, 77]. The involvement of a methodologist was clearly reported in nine guidelines (11.8% of the cases) [15, 26, 30–32, 40, 42]. In the specific subgroup of procedural guidelines (*n* = 22), key technical or interpretation procedures were clearly and exhaustively specified in ten guidelines (45.5%) [8, 12, 18, 61, 65, 68, 69, 73, 74] and partially reported in 11 guidelines (58.8%) [10, 17, 18, 62–64, 66, 67, 71, 72, 75]. In the specific subgroup of guidelines dealing both with clinical and procedural topics (*n* = 7), key technical or interpretation procedures were partially reported in 57.1% of cases [10, 78–80] and not reported in the remaining 42.9% [19, 76, 77]. The differences in methodology between the two continents with the highest number of official documents (Europe and North America) is provided in Table 3.

**Table 2:** Methods for the collection and use of evidence

Methods surveyed	Guidelines for Clinical N (%)	Guidelines for procedures N (%)	Guidelines for Both N (%)
Number of guidelines	47	22	7
Evidence based			
• Systematic review • Non-systematic review • Mixed • Not reported	5 (10.6%) 22 (46.9%) 11 (23.4%) 9 (19.1%)	1 (5.9%) 12 (58.8%) 1 (5.9%) 8 (29.4%)	0 (0%) 2 (28.6%) 3 (42.8%) 2 (28.6%)
Search strategies			
• Clearly reported • Not reported • Not applicable	19 (40.4%) 27 (57.4%) 1 (2.2%)	9 (41%) 12 (55%) 1 (4%)	0 (0%) 7 (100%) 0 (0%)
Strength of recommendation			
• Reported • Not reported • Not applicable	19 (40.4%) 27 (57.4%) 1 (2.2%)	1 (4%) 20 (92%) 1 (4%)	0 (0%) 7 (100%) 0 (0%)
External review			
• Clearly reported • Not reported • Not applicable	16 (34%) 30 (63.8%) 1 (2.2%)	1 (4%) 20 (92%) 1 (4%)	1 (14.3%) 6 (85.7%) 0 (0%)
Involvement of methodologist			
• Clearly reported • Not reported • Not applicable	9 (19.1%) 37 (78.7%) 1 (2.2%)	0 (0%) 21 (96%) 1(4%)	0 (0%) 7 (100%) 0 (0%)

**Table 3:** Overview of methodology of the two continents with the highest number of official documents

	PET(CT)	SPECT(CT)	PET and SPECT	Original	Cumulated citation	Key procedure fully provided
Europe	12/26 (46%)	4/26 (15%)	10/26 (39%)	24/26 (92%)	2338	8/26 (31%)
North America	8/15 (53%)	3/15 (20%)	4/15 (27%)	9/15 (60%)	1407	3/15 (20%)
	Systematic review	Strategy reported	Recommendation(s) reported	External review	Methodologist	
Europe	1/26 (3.8%)	7/26 (27%)	4/26 (15%)	4/26 (15%)	2/26 (7.7%)	
North America	2/15 (13%)	12/15 (80%)	9/15 (60%)	10/15 (67%)	4/15 (27%)	

## Discussion

The aim of our study was to evaluate the level of evidence of expert recommendations and guidelines for clinical indications and procedurals in hybrid cardiovascular nuclear imaging published for the last ten years. Clinical and procedural guidelines are relevant documents both in hybrid cardiovascular imaging and in other fields, aiming to improve the quality of care while reducing variability in clinical practice and containing healthcare costs [81].

First, we observed an increasing number of guidelines on hybrid cardiovascular nuclear imaging in recent years. This is expected and in line with the demonstration that the number of clinical practice guidelines produced for healthcare has risen exponentially in the last 20 years [81]. Two recent clinical guidelines of the European Society of Cardiology assigned a high class of recommendation and high level of evidence for our nuclear medicine techniques: IB for [^18^F]FDG in infective endocarditis and IB for [^99m^Tc]-bone seeking agents in patients suspected of cardiac amyloidosis [24, 33]. Of note, amyloidosis concerned the majority of the guidelines retrieved during the period 2020–2023. This could be explained by the FDA clearance of the first anti-amyloid drugs in 2019 and related treatment strategy issues, which potentially stimulated the interest for patient screening in this field.

Second, we observed several heterogeneous characteristics in the selected guidelines about type of guidelines, disease and hybrid imaging evaluated, radiotracers, targeted population, guideline developing organizations, and guideline citations. This heterogeneity was expected since the only common denominator of the selected guidelines was hybrid cardiovascular imaging.

The most important aim of our study was the quality assessment of the included guidelines on cardiovascular hybrid imaging focusing on use of evidence, search strategy, strength of recommendations, external review, and methodology.

About the use of the evidence, notably, only few selected guidelines are evidence-based documents based on systematic reviews. Evidence-based guidelines are an important tool for healthcare professionals to make informed decisions about patient care. Compared to non-evidence-based guidelines, they can help to minimize bias and enhance the quality and consistency of clinical practice or public health policy. Evidence-based guidelines are developed using a rigorous process that involves identifying the best available evidence, evaluating its quality, and synthesizing it into recommendations for clinical practice or public health policy. Translating evidence into practice can not only improve outcomes and quality of life for patients but also improve productivity and reduce healthcare costs [82]. Beyond cardiovascular hybrid imaging, not evidence-based guidelines are frequent encountered for other pathologies. Using non-systematic methods in clinical practice and procedural guidelines compromises the validity and reliability of the evidence used to inform guideline recommendations, leading potentially to misleading and untrustworthy results [83].

Search strategies are important in evidence-based guidelines and systematic reviews because they help researchers identify all relevant studies that meet the inclusion criteria, while minimizing the risk of missing important studies [84]. A search strategy was reported only in about half of the selected guidelines on cardiovascular hybrid imaging. As a consequence, the transparency and reproducibility of guidelines are compromised.

In most of the included guidelines, strength of recommendations is not reported. The strength of recommendations in guidelines is a measure of its confidence in the effectiveness of an intervention or a diagnostic method. The strength of recommendation is a grading scale that is used to rate the quality, quantity, and consistency of evidence. Unfortunately, many guidelines are inconsistent in rating the quality of evidence and the strength of recommendations. The GRADE system is a consensus on rating quality of evidence and strength of recommendations, which is increasingly being adopted by organizations worldwide [85, 86].

External reviews of guidelines are a way to ensure that the guidelines are of high quality and based on the best available evidence. External reviews can be conducted by independent experts or organizations, and they can provide valuable feedback on the content, format, and implementation of guidelines. External reviewers should comprise a full spectrum of relevant stakeholders, including scientific and clinical experts, organizations, agencies, patients, and representatives of the public [6].

Unfortunately, only a limited number of guidelines in our study included an external review.

Finally, a methodologist in the context of clinical practice guidelines is a professional who specializes in the development and implementation of clinical practice guidelines. Methodologists are responsible for ensuring that guidelines are based on the best available evidence and that they are developed using a rigorous and transparent process, working closely with the guideline development groups. Overall, a thorough methodological approach is needed for developing, reporting, and assessing evidence-based clinical practice guidelines [87]. Only few of the included guidelines on hybrid cardiovascular imaging included a methodologist.

Currently, there remains ongoing inconsistency in quality of clinical practice guidelines and procedural guidelines on hybrid cardiovascular imaging. Of note, our results are in line with two recent similar methodological works applied in the general field of PET imaging [7, 88]. The excessive number of low-quality guidelines also wastes resources and the efforts of care providers who rely on guidelines to inform their decision-making and clinical practice [81]. To address this issue, significant efforts are mandatory to improve the methodological quality of guidelines and establish a standardized approach to develop evidence-based guidelines in hybrid cardiovascular imaging as in other medical fields. Incorporation of our (hybrid) nuclear medicine imaging techniques in (clinical) guidelines is pivotal, for visibility and clinical use. Because the role and support from the international consortiums is essential, the EANM has recently launched a dedicated Guidelines and Publications Council to further improve the development of guidelines and ensure high-quality standards.

## Conclusions

The use of evidence procedures for evidence-based developing cardiovascular hybrid imaging recommendation guidelines is currently suboptimal, highlighting the need for more standardized methodological procedures.

## Declarations

### Guarantor

The scientific guarantor of this publication is Professor Riemer H.J.A Slart, from Department of Nuclear Medicine, University Medical Center Groningen, Netherland.

### Statistics and biometry

Some of the authors (GT, FLB) have significant statistical expertise.

### Study subjects or cohorts overlap

No study subject or cohort has been previously reported.

### Methodology

• Retrospective

• Cross-sectional study

• Literature survey
